# Development of advanced bioinformatic profiles to improve the detection and functional understanding of fungal acid phosphatases

**DOI:** 10.1128/aem.02106-25

**Published:** 2026-06-12

**Authors:** Tamara Gómez-Gallego, Zulema Udaondo, Rocio Palacios-Ferrer, Luis Díaz-Martínez, Juan L. Ramos

**Affiliations:** 1 Department of Environmental Protection, Estación Experimental del Zaidín, Consejo Superior de Investigaciones Científicas (CSIC) 73025, Granada, Spain; 2 Department of Microbial Biotechnology, National Center for Biotechnology, Consejo Superior de Investigaciones Científicas (CSIC), Campus de la Universidad Autónoma de Madrid 16379, Madrid, Spain; 3 Programa de Doctorado en Bioquímica y Biología Molecular, Universidad de Granada 16741, Granada, Spain; 4 Departamento de Microbiología, Instituto de Hortofruticultura Subtropical y Mediterránea ‘La Mayora’, Universidad de Málaga-Consejo Superior de Investigaciones Científicas (IHSM-UMA-CSIC), Universidad de Málaga 518806 , Málaga, Spain; Royal Botanic Gardens Kew, Surrey, United Kingdom

**Keywords:** phosphatase, phosphate availability, phosphorus: soil sustainability, soil preservation, phosphorus cycle, PROSITE protein profiles, agro-systems, soil enzymes

## Abstract

We have retrieved approximately 9,000 protein sequences annotated as fungal acid phosphatase or phytase from the UniProtKB database. Following stringent quality filtering, a curated dataset comprising 3,058 high-confidence sequences was assembled. Phylogenetic analysis resolved these enzymes into eight distinct clades, representing distinct groups of fungal acid phosphatases: purple acid phosphatases, phytases, and groups containing both phytases and acid phosphatases annotations. Based on this classification, we have developed three representative protein profiles referred to as Prf-A-Fungal_phos, Prf-B-Fungal_phos, and Prf-C-Fungal_phos, each designed to capture the phylogenetic and functional diversity of these enzyme families. Heat-map analyses confirmed the breadth and high specificity of these profiles. Application of these profiles to public protein and metagenomic databases enabled the identification of hundreds of previously uncharacterized fungal proteins, with a broad taxonomic distribution and notable prevalence in the *Ascomycota* and *Basidiomycota* phyla. Functional validation through heterologous expression of selected candidates in *Saccharomyces cerevisiae* confirmed their phosphatase activity, supporting the accuracy of the *in silico* predictions. By integrating large-scale bioinformatics with experimental validation, this study provides robust tools for the discovery of novel fungal phosphatases and for investigation of their ecological roles in nutrient-limited environments.

**IMPORTANCE:**

Fungal acid phosphatases are critical enzymes in global phosphorus cycling, yet no dedicated bioinformatic tools exist to comprehensively identify and classify them across fungal diversity. Here, we present the first PROSITE generalized profiles specific to fungal acid phosphatases, derived from a curated data set of over 3,000 high-confidence sequences spanning eight phylogenetic groups. These profiles exhibit high specificity and sensitivity, enabling the detection of hundreds of previously uncharacterized proteins from public protein databases. Experimental expression of representative candidates in *Saccharomyces cerevisiae* confirmed their phosphatase activity, validating our *in silico* predictions. By bridging large-scale bioinformatics with functional validation, this study delivers robust resources to uncover novel fungal phosphatases and to explore their ecological roles in nutrient-limited environments. The developed profiles will advance metagenomic annotation, support soil and environmental microbiology research, and foster biotechnological innovation in sustainable phosphorus management.

## INTRODUCTION

Phosphorus (P) is an essential macronutrient required for the synthesis of key cellular components, including nucleic acids, phospholipids, and energy carriers. such as pyrophosphate, ADP and ATP, as well as a wide range of organophosphorus compounds vital to all living organisms (1). Although phosphorus is abundant in the Earth’s crust, it is predominantly present in insoluble forms, making it largely unavailable to plants, macroalgae, and microorganisms (2). Therefore, the global biogeochemical cycling of P is crucial for ecosystem productivity and is closely linked to carbon and nitrogen cycles (3, 4). Unlike carbon and nitrogen, the phosphorus cycle lacks a significant gaseous phase and enters ecosystems mainly through rock weathering and, to a lesser extent, dust deposition (2, 5), rendering P availability a limiting factor in many terrestrial and aquatic ecosystems (6). Phosphorus is also critical for crop productivity, yet its availability in many soils is limited and depends on finite phosphate rock reserves, raising concerns about long-term sustainability (4, 7, 8). Recent genome-resolved metagenomic studies have further highlighted that soil microbiomes deploy distinct phosphorus acquisition strategies and that phosphate-solubilizing bacteria (PSB) in agricultural soils possess specific genetic traits linked to P solubilization (9, 10).

Microorganisms, particularly bacteria and fungi, play a central role in mobilizing phosphorus from organic and inorganic sources (11). Inorganic phosphates, such as aluminum, calcium, and iron phosphates, are mainly solubilized through the microbial production of organic acids (e.g., gluconic, oxalic, lactic, malonic), which acidify the local environment and enhance P solubilization (9, 10). In contrast, the mineralization of organic phosphorus compounds depends on the secretion of phosphatase enzymes by microbes and plants that hydrolyze these compounds, releasing orthophosphate (12, 13).

Phosphatases are broadly categorized into two major groups based on their optimal pH (14, 15): (i) alkaline phosphatases (EC 3.1.3.1), which function preferentially at pH values of between 7.5 and 8.5, and are predominantly of microbial origin (12, 16). These enzymes, encoded by gene families, such as *phoA*, *phoD*, *phoX*, and *psiP1*, in bacteria, and *pho8*, *xpp1*, *ssu78*, *siw114*, and *alp1* in fungi, are widespread in soils and marine environments (17–22). They play a dominant role in organic P mobilization in alkaline soils. Although alkaline phosphatases have historically been considered less relevant in acidic environments, recent studies have documented their abundance in forest and agricultural soils (2, 23–25), and (ii) acid phosphatases (EC 3.1.3.2), which function optimally at lower pH values, typically between 5 and 6. These enzymes, commonly released by plants and microbes (26), hydrolyze phosphoesters and phosphoanhydrides. Their ecological importance has been highlighted by recent studies (23, 25, 27), which demonstrate their key role in P cycling in acidic soils, where they usually exhibit high substrate affinity. Genomic and metagenomic analyses further support their widespread distribution, revealing the dominance of *phoC* family members (13, 18, 28).

Soil phosphatase activity is influenced by environmental factors such as total nitrogen content, precipitation, temperature, and agricultural management practices, including reduced tillage (23, 25, 27, 29, 30). A recent meta-analysis by Rocabruna et al. (31) demonstrated that alkaline and acid phosphatases respond differentially to land management practices: acid phosphatase activity was stimulated by organic fertilization combined with crop rotation or irrigation, whereas alkaline phosphatase activity responded more strongly to the combination of organic fertilization and tillage reduction. Phosphatases facilitate organic matter decomposition and improve plant nutrient uptake, highlighting the contribution of these enzymes to soil fertility. However, measured soil enzymatic activities represent a heterogeneous pool of enzymes, including free enzymes and those adsorbed onto soil particles and clays, complicating the attribution of activity to specific microbial guilds or plants.

To overcome these limitations, bioinformatic analyses provide a powerful approach for identifying untapped phosphatases (and other enzymes) directly from genomic and metagenomic data. Such analyses require the use of updated and optimized bioinformatic tools, including specialized protein profiles, to accurately detect and classify functionally relevant enzymes in complex environmental databases (28, 32).

In a previous study, we analyzed bacterial acid phosphatases and, using a curated data set of 3,741 sequences, identified three main groups, B, C, and generic acid phosphatases (GAPs, Group A) (28). Phylogenetic analysis revealed that groups A and C are more closely related to each other than to group B. The construction of group-specific protein profiles enabled the identification of numerous previously uncharacterized bacterial acid phosphatases.

Given the high abundance of both phosphate-solubilizing bacteria and phosphorus-mobilizing fungi in soils, we have now extended this approach to fungal acid phosphatases and constructed a phylogenetic tree that has revealed eight distinct groups within the fungal kingdom.

At the biochemical level, two major and well-characterized classes of fungal acid phosphatases have been described. Histidine acid phosphatases (HAPs) are characterized by a conserved catalytic histidine residue and the presence of an RHGXRXP motif. Members of this family typically exhibit broad substrate specificity and are active across a range of acidic pH values. A specialized subgroup within the HAP family, phytases (EC 3.1.3.8, EC 3.1.3.26), hydrolyze phytic acid (phytate) (myo-inositol hexakisphosphate), a major organic P reserve in seeds, releasing phosphate under both acidic and alkaline conditions (33). The second major class comprises purple acid phosphatase (PAPs), a distinct group of secreted metallophosphoesterases characterized by a Fe(III)-tyrosinated active center that confers a characteristic purple color. This group of fungal acid phosphatases shares a conserved active site motif with plant and mammalian PAPs, but lacks the RHGXRXP motif found in histidine acid phosphatases, reflecting a distinct catalytic mechanism (34).

To obtain a comprehensive view of untapped fungal acid phosphatases in soils and other ecosytems, we developed three generalized PROSITE profiles for fungal acid phosphatases: one specific to Group I, another targeting Group II, and a third covering Groups III–VIII. These profiles provide a valuable resource for integrating high-throughput sequencing with field data, improving models of phosphorus cycling, and informing the design of microbial-based biofertilizers.

## MATERIALS AND METHODS

### Phylogenetic tree construction

Protein sequences were retrieved from UniProtKB database (https://www.uniprot.org/) using advanced searches, filtering for fungal sequences annotated with either “acid phosphatase” or “phytase” in the protein name. This search yielded 5,269 sequences annotated as “acid phosphatase” and 3,590 sequences annotated as “phytase,” resulting in a total of 8,859 protein sequences (data as of 6 May 2023). Sequences were filtered based on length (mean ± 1 standard deviation), and outliers that were significantly longer or shorter were removed. Redundant sequences with 100% identity were discarded using EMBOSS Skipredundant software v6.6 (35). After manual curation, the data set was reduced to 4,004 sequences. These sequences were aligned using MUSCLE v3.7 software with the parameters --maxiters 1,000 (36). Highly divergent sequences were removed to improve alignment quality. The curated data set comprises 3,058 proteins from 810 species and 314 genera (Table S2), spanning two fungal phyla. The final set of 3,058 sequences was retained, re-aligned as detailed above, and used to construct a phylogenetic tree using the IQ-TREE software v1.6.12 (37). The following parameters were used for tree construction: -nt AUTO, -bb 1,000; -m TESTMERGE; and -safe, allowing the exploration of phylogenetic relationship among sequences. The maximum likelihood tree was constructed using the WAG+I+G4 model, identified as the best-fit evolutionary model by ModelFinder (38). The phylogenetic tree was visualized using the Interactive Tree of Life (iTOL) suite, software v4 (39).

### Protein profile construction

As an initial step toward creating unique PROSITE generalized profiles, we defined a seed of protein sequences for each enzyme group. The selection of these proteins significantly influences the sensitivity and overall quality of the resulting profiles. For this purpose, full sequences from each group of enzymes were aligned separately using MUSCLE v3.7, as detailed previously. Divergent sequences were further filtered if necessary to enhance alignment consistency. We then used the pfw function from the PFTOOLs v2.3. package to calculate the weights of individual protein sequences within each multiple sequence alignment. The weighted alignments were then converted into generalized profiles using pfmake, applying the parameters: −2b; -H2.0, with BLOSUM45 as the substitution score matrix. To calibrate the profiles, we used pfsearch and pfscale from the PFTOOLs suite. The calibration database was constructed by filtering fungal proteins from the entire protein Swiss-Prot database (release date 23 February 2022), resulting in 34,929 sequences, which were randomly shuffled using a sliding window of 20 residues (-kmer 20) using the fasta-shuffle-letters algorithm from MEME suite v5.1.1. After obtaining the calibrated profiles, each profile was compared against the complete database of fungal phosphatases of the phylogenetic tree (3,058 sequences) using pfsearch with parameters –z –f (40) to verify its specificity and ensure it recognized only sequences from its corresponding group.

### Validation of fungal phosphatase profiles in databases

To further validate protein profiles, each profile was also tested against different protein databases using pfsearch, as detailed above. The resulting match lists were analyzed to warrant satisfactory sensitivity. The databases used were (i) the complete data set of non-curated fungal phosphatase sequences (8,859 protein sequences), (ii) fungal protein sequences annotated as “acid phosphatase” or “phytase” in the UniProtKB database (20,050 protein sequences, release date 21 November 2024), (iii) the full collection of fungal protein sequences in the UniProtKB database (19,274,552 sequences, release date 21 November 2024), and a UniRef90 database containing 29,638,836 clusters of sequences filtered using the following criteria: “Taxonomy: 4751”, which corresponds to Fungi; and “Cluster name: Hypothetical protein OR Unknown protein OR Uncharacterized protein”. The “Cluster” name corresponds to the protein name in UniRef90.

### Cloning and expression of fungal phosphatases in yeast

As a proof of concept to empirically validate fungal phosphatase profiles, the non-revised sequences with the highest Z-score within each profile were synthesized *in vitro* using codon optimization for *Saccharomyces cerevisiae*.

The selected sequences were (i) a putative purple acid phosphatase from *Hyaloscypha variabilis* (UniProtKB ID A0A2J6S554) for Prf-A-Fungal_phos, herein referred to as HvPhyA; (ii) a putative acid phosphatase/3-phytase from *Verticillium alfalfae* (UniProtKB ID A0A1D9Q6T7) for Prf-B-Fungal_phos, referred to as ScPhyB; (iii) a putative acid phosphatase from *Ascochyta rabiei* (UniProtKB ID A0A162Z3M2) for Prf-C-Fungal_phos, referred to as DrPhoC. The corresponding DNA sequences were codon-optimized for expression in *S. cerevisiae*, synthesized *in vitro* (GenScript), and cloned into the yeast expression vector pDR196 or pYES2 (see the supplemental methods) between the PstI and SalI sites. All constructs were verified by sequencing. The BY4741 strain (*MATa his3Δ1 leu2Δ0 met15Δ0 ura3Δ0*) (https://www.yeastgenome.org/strain/by4741) (41, 42) was transformed using the lithium acetate-based method (43), with either the recombinant constructs or an empty pDR196 vector. Transformants were selected on synthetic dextrose (SD) minimal medium based on uracil autotrophy. All primers used in this study are listed in Table S1.

### Phosphatase assay in yeast cell cultures

Yeast transformants were grown for 24 h in SD-selective medium without uracil. In the case of strains expressed using the cloning vector pDR, yeast transformants were grown for 24 h in selective SD medium without uracil and supplemented with glucose. When the pYES2 vector was used, transformants were grown in selective SD medium lacking uracil supplemented with galactose to induce the promoter. Phosphatase and phytase activities were assayed in cell-free extracts. For this, 20 mL of culture was centrifuged at 10,000 × *g* for 10 min, the supernatant was discarded, and the pellet was frozen at −80°C. Pellets were resuspended in 3 mL of HAM buffer (40 mM HEPES, 40 mM acetic acid, and 40 mM MES, pH 5.5) (44), and cell lysis was achieved using three passes through a French press at a pressure of 1,000 p.s.i. The lysates were centrifuged as above, and the resulting pellet was resuspended in 1000 µL of HAM buffer. Standard acid phosphatase activity was measured by quantifying the amount of *p*-nitrophenol (PNP) produced from 4-nitrophenyl phosphate (pNPP) at 405 nm using an extinction coefficient *ε* = 1.8 × 10^4^ M^−1^ cm^−1^. Briefly, 100 µL of resuspended pellet or supernatant were mixed with 20 µL of pNPP (0.115 M) and incubated for 15 min at 37°C in HAM buffer at pH 5.5. The reaction was stopped by adding 100 µL of NaOH and 0.8 mL of distilled water (44). The amount of PNP produced was quantified spectrophotometrically at 405 nm using a Tecan Sunrise plate reader (Tecan Austria GmbH). When indicated the incubation temperature was 20°C, 30°C, 40°C, 50°C, 60°C, and 70°C. Since HAM buffer covers the range 4.0–8.5, assays were also performed at pH 4.5, 5.5, 6.5, 7.5, and 8.5 when indicated.

### Statistical analyses and heatmap representation

All the statistical analyses were performed using the sequence similarities, pairwise sequence similarities were calculated using global pairwise alignment with the align.globalxx function from the Bio.pairwise2 module, which computes percent similarity based on the number of identical matches in the aligned region. The heatmap was constructed using the ComplexHeatmap package (45), with color gradients representing similarity values from 0% (blue) to 100% (red) via a midpoint of 50% (white), scaled using colorRamp2 from the circlize package (46). Group-level annotations were added as color-coded sidebars using a categorical color palette from RColorBrewer enabling easy visual discrimination among groups.

## RESULTS AND DISCUSSION

**Fig 1:**
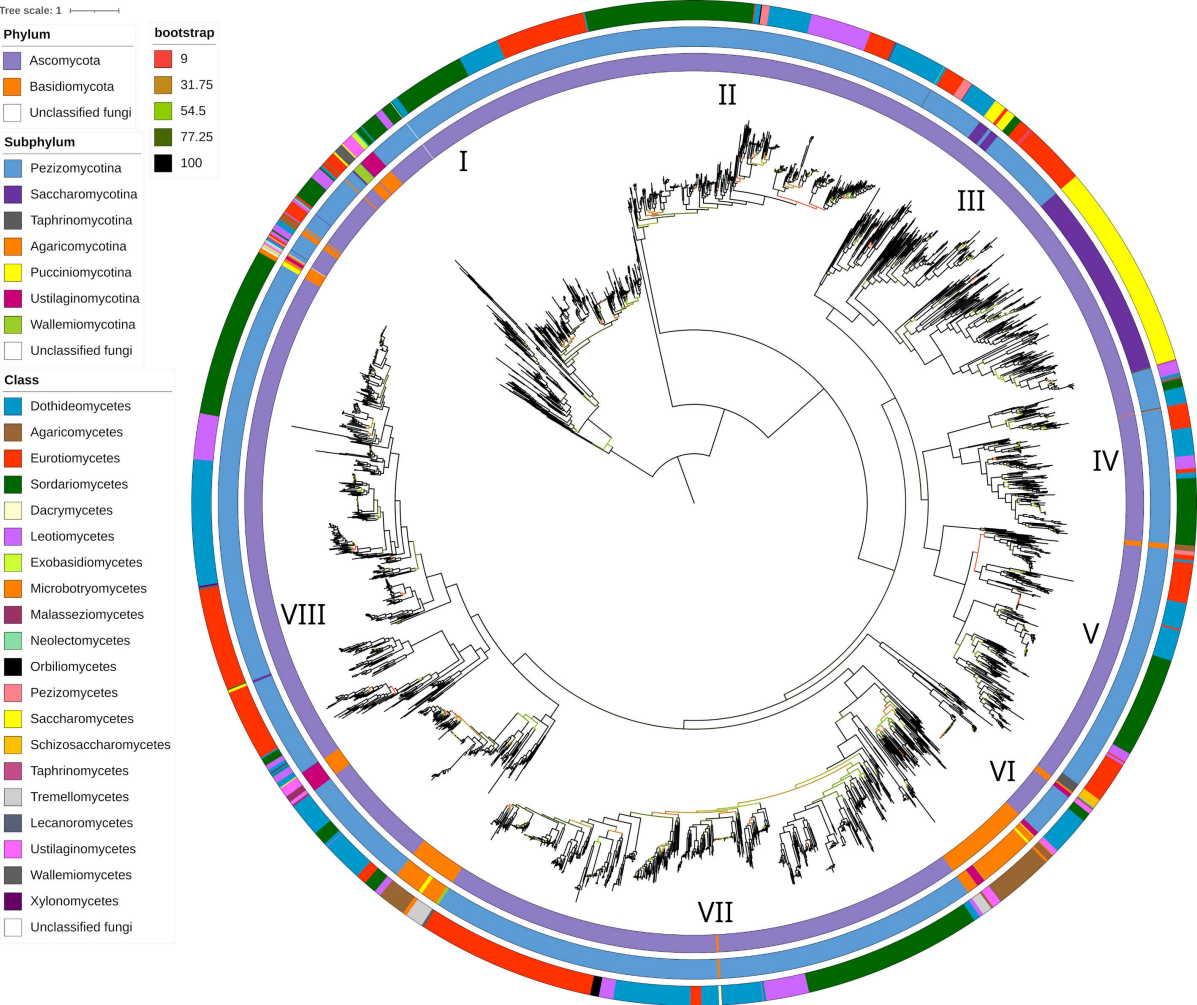
Maximum likelihood phylogenetic tree of fungal acid phosphatase and phytase proteins. This analysis compares the phylogenic relationships among 3,058 fungal amino acid sequences annotated as “acid phosphatase” or “phytase” in the UniProtKB database. The tree was constructed using the maximum likelihood method with the WAG+I+G4 model of amino acid sequence evolution in IQ-TREE. The tree was rooted using the midpoint method. Branch colors represent confidence intervals based on bootstrapp analyses with 1,000 replicates, as indicated in the figure legend. Taxonomic labels were added to visualize the taxonomic distribution with concentric circles representing different taxonomic levels from phylum (inner ring) to class (outer ring). Detailed metadata for protein sequences are provided in Table S2.

We have identified eight major clades of protein sequences based on the constructed phylogenetic tree (Fig. 1), and further characterized them using information from the UniProtKB database. A summary of the sequence details is presented in Table 1, with extended raw data available in Table S2. The curated data set comprises 3,058 high-confidence sequences representing 810 fungal species and 314 genera across two major phyla. Although public databases are known to be biased toward Ascomycota, this data set captures broad phylogenetic diversity within the fungal kingdom.

**TABLE 1:** Detailed information of major groups of fungal phosphatase sequences identified in the phylogenetic tree^*a*^

Group	Seq	Protein family	EC no.	GO ID	InterPro	Pfam
I	403	Metallophosphoesterase superfamily; purple acid phosphatase family	Acid phosphatase, 3.1.3.2	GO:0003993 GO:0046872	IPR004843 (100%); IPR029052 (100%); IPR039331 (100%); IPR008963 (100%); IPR041792 (99.75%); IPR025733 (99.26%); IPR015914 (98.01%);	PF00149 (100%) PF14008 (99.25%) PF16656 (98.01%)
II	360	HAP family	3-Phytase, 3.1.3.8	GO:0016158 GO:0016791	IPR000560 (100%); IPR029033 (100%); IPR033379 (99.72%)	PF00328 (100%)
III	370	HAP family	Acid phosphatase, 3.1.3.2; 3-Phytase, 3.1.3.8	GO:0016158 GO:0008707 GO:0016791 GO:0003993 GO:0017111 GO:0047429	IPR000560 (99.73%); IPR029033 (99.73%); IPR016274 (92.97%); IPR033379 (87.84%)	PF00328 (99.73%)
IV	183	HAP family	Acid phosphatase 3.1.3.2	GO:0003993 GO:0016791	IPR000560 (100%); IPR029033 (100%) IPR016274 (100%)	PF00328 (100%)
V	266	HAP family	4-Nitrophenylphosphatase, 3.1.3.41; acid phosphatase, 3.1.3.2; 3-Phytase, 3.1.3.8	GO:0016158 GO:0016791 GO:0003993	IPR000560 (97.74%); IPR029033 (97.74%); IPR016274 (91.73%); IPR033379 (85.71%)	PF00328 (97.74%)
VI	64	HAP family	Acid phosphatase, 3.1.3.2; 3-phytase, 3.1.3.8	GO:0016158 GO:0016791 GO:0003993	IPR000560 (100%); IPR029033 (100%); IPR016274 (100%); IPR033379 (100%)	PF00328 (100%)
VII	669	HAP family	3-Phytase, 3.1.3.8; 4-phytase, 3.1.3.26	GO:0016158 GO:0008707 GO:0016791 GO:0003993	IPR000560 (100%); IPR029033 (100%); IPR033379 (98.51%); IPR016274 (86.40%)	PF00328 (100%)
VIII	743	HAP family	3-Phytase, 3.1.3.8	GO:0016158 GO:0003993 GO:0016791	IPR000560 (100%); IPR029033 (100%); IPR033379 (88.83%); IPR016274 (9.42%)	PF00328 (100%)

^*a*^ Cross-references of annotated domains available in UniProtKB from InterPro and pfam databases are also provided with entry codes exclusive to a group highlighted in bold; their percentage of occurrence in each group is indicated in parentheses. Gene ontology (GO) IDs, molecular function: acid phosphatase activity, GO:0003993; metal ion binding, GO:0046872; phosphatase activity, GO:0016791; nucleoside-triphosphate diphosphatase activity, GO:0047429; nucleoside-triphosphatase activity, GO:0017111; 3-phytase activity, GO:0016158; 4-phytase activity, GO:0008707; InterPro codes: calcineurin-like phosphoesterase domain, ApaH type, IPR004843; metallo-dependent phosphatase-like, IPR029052; purple acid phosphatase-like, IPR039331; purple acid phosphatase-like N-terminal, IPR008963; purple acid phosphatase, metallophosphatase domain, IPR041792; iron/zinc purple acid phosphatase-like C-terminal domain, IPR025733; purple acid phosphatase, N-terminal, IPR015914; histidine phosphatase superfamily, clade-2, IPR000560; histidine phosphatase superfamily, IPR029033; HAP active site, IPR033379; HAP, eukaryotic, IPR016274. Pfam codes: calcineurin-like phosphoesterase, PF00149; iron/zinc purple acid phosphatase-like protein C, PF14008; purple acid phosphatase, N-terminal domain, PF16656; histidine phosphatase superfamily (branch 2), PF00328. Extended information on protein sequences is provided in Table S2.

The phylogenetic analyses resolved proteins annotated as fungal purple acid phosphatases (PAPs) (Group I), acid phosphatases (Group IV), phytases (Groups II, VII and VIII), and groups containing both phytases and acid phosphatases (Groups III, V, and VI). Remarkably, most phytase-containing groups included sequences annotated with Gene Ontology (GO) term GO:0016158, which corresponds to enzymes with 3-phytase activity, while Groups III and VII contained protein sequences annotated with GO:0008707, characteristic of enzymes with 4-phytase activity.

A comprehensive analysis of signal peptide predictions across the curated data set revealed that 1,556 of the 3,058 sequences (50.9%) contain a predicted N-terminal secretion signal. The proportion of predicted secreted proteins was nearly identical in Ascomycota (50.9%) and Basidiomycota (50.6%), indicating that predicted secretion occurs at similar frequencies in both phyla. However, marked differences were observed among phylogenetic groups. Groups I, IV, V and VI exhibited high proportions of predicted signal peptides (81%–88%), consistent with predominantly extracellular roles, whereas Group VII (29%) and particularly Group II (0%) were largely devoid of signal peptides. These patterns suggest functional diversification among fungal phosphatases clades, with certain groups likely to be specialized for extracellular phosphorus mobilization and others potentially fulfilling intracellular or periplasmic roles.

We found that Group I, the most evolutionarily distinct clade, comprised 403 protein sequences (13.18% of the data set), all annotated as PAPs. These enzymes belong to the metallophosphoesterase superfamily and are associated with EC 3.1.3.2 and GO terms GO:0003993 or GO:0046872 (47). The clear phylogenetic separation of Group I sequences observed in the phylogenetic tree supports the early divergence of PAPs from the other fungal acid phosphatases. As shown in Table S2, about 85% of the proteins in this group contain a predicted signal peptide (15 to 27 amino acids in length), consistent with their extracellular role (48). This group is predominantly represented by enzymes from *Aspergillus*, *Talaromyces*, and *Trichoderma*, taxa widely known for their roles in nutrient mobilization and phosphate solubilization in low-P environments, as well as for their ability to colonize plant and animal hosts.

Group II included 360 fungal protein sequences (11.77% of the total number of sequences). These sequences were mostly annotated as 3-phytases within the HAP family, associated with EC 3.1.3.8 and GO terms GO:0016158 and GO:0016791 (49). Taxonomically, this group is diverse but particularly enriched in genera, such as *Colletotrichum*, *Fusarium*, and *Verticillium*, many of which are plant-associated, including phytopathogens and endophytes.

Group III consisted of 370 sequences (12.10%), annotated as either acid phosphatase or phytase. These belong to the HAP family, with EC 3.1.3.2 or EC 3.1.3.8, and GO terms GO:0016158, GO:0008707, GO:0016791 or GO:0003993. This group shows broad functional annotation and included members of Saccharomycetales such as *Candida*, *Pichia*, and *Saccharomyces*, suggesting a strong representation of yeasts, many of which are associated with human hosts, fermentation, or opportunistic pathogens.

Group IV includes 183 sequences, annotated solely as acid phosphatases (5.98% of the total number of sequences), within the HAP family, EC 3.1.3.2, and with GO terms GO:0,016,791 or GO:0,003,993. This group is taxonomically diverse, including both filamentous fungi and yeasts, such as *Kluyveromyces*, *Candida* and *Yarrowia,* spanning both commensal yeast and saprotrophic fungi, reflecting functional diversity across ecological strategies.

Group V comprises 266 sequences (8.70% of the total number of sequences), representing a group containing enzymes annotated as putative acid phosphatase and phytase within the HAP family, with EC 3.1.3.2 or EC 3.1.3.8 and GO terms GO:0016158, GO:0016791 or GO:0003993. Members of this group included representatives from *Beauveria*, *Trichoderma*, and *Thermomyces*, which are general known for their entomopathogenic activity and thermotolerance.

Group VI, the smallest group with 64 protein sequences (2.09%), also contained proteins annotated as either acid phosphatase or phytase within the HAP family, with EC 3.1.3.8 or EC 3.1.3.2 and with any of the following GO terms: GO:0016158, GO:0016791 or GO:0003993. Organisms within this group are mostly filamentous saprotrophs with thermotolerant or stress-resistant traits, such as *Thermoascus*, *Myceliophthora*, and *Talaromyces,* known for their thermophilic or stress-resistant traits and roles in cellulose degradation and composting (50).

Group VII contained 669 protein sequences (21.88%), annotated mainly as phytases from the HAP family, with EC 3.1.3.8 or EC 3.1.3.26 and GO terms: GO:0016158, GO:0008707, GO:0016791 or GO:0003993. This group was taxonomically broad, including thermophilic and soil fungi such as *Rasamsonia*, *Rhizomucor*, and *Myceliophthora*, often found in compost and decaying plant material. Finally, Group VIII, the largest group, comprised 743 protein sequences, all within the HAP family (24.30%), and associated with EC 3.1.3.8 and GO terms GO:0016158 or GO:0016791 or GO:0003993. This group encompassed a broad diversity of fungi across various lifestyles, including model organisms, opportunistic pathogens, and symbionts such as *Neurospora*, *Candida*, *Aspergillus*, and *Pichia*, underscoring its functional and evolutionary versatility. Together, these eight groups underscore the evolutionary complexity and functional diversity of fungal acid phosphatases, reflecting diverse ecological strategies and environmental adaptations.

**Fig 2:**
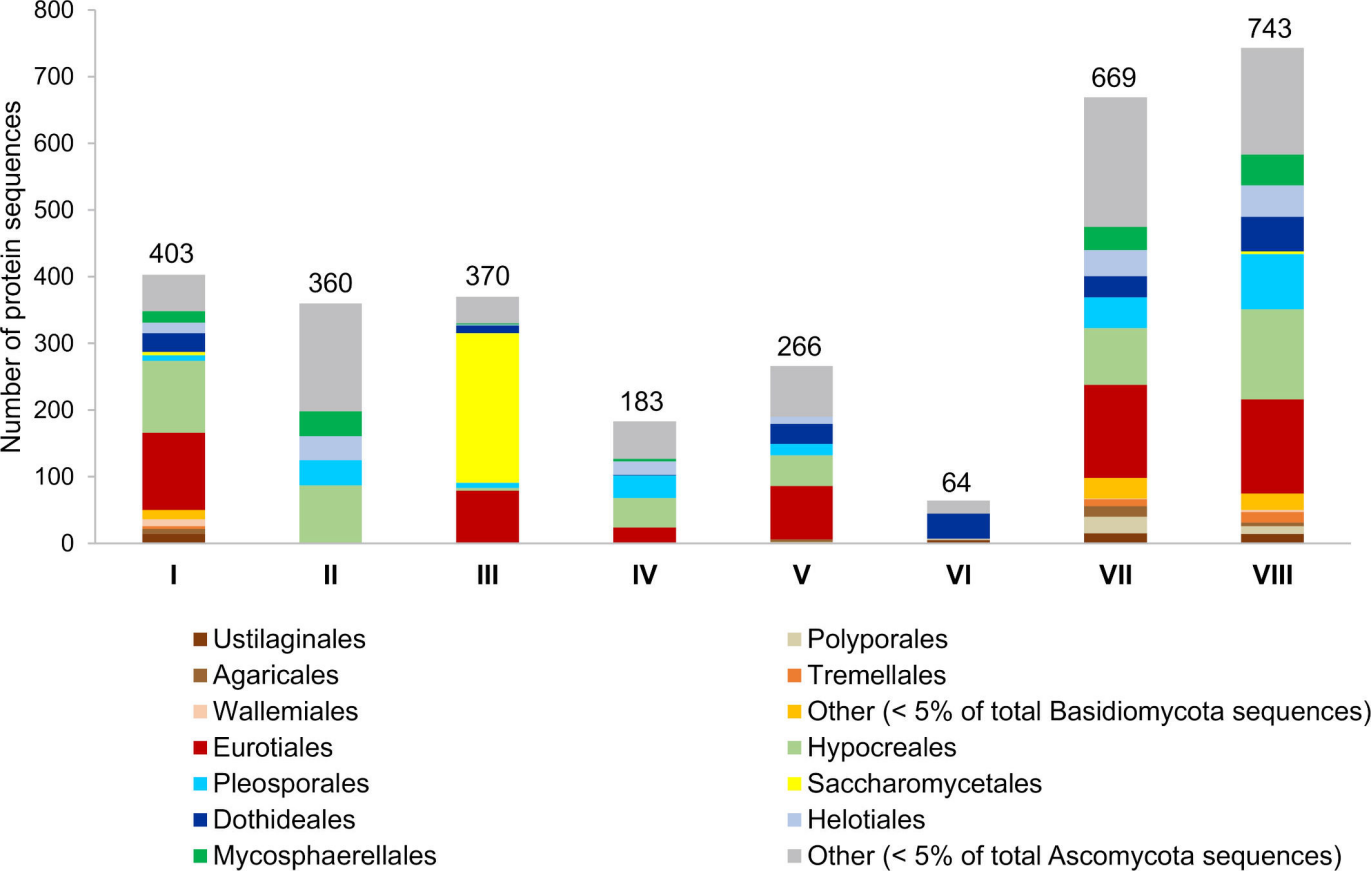
Taxonomic distribution of protein sequences across phosphatase groups. The number of sequences per group is shown at the order level based on the data set used to construct the phylogenetic tree. In total, 63 fungal orders were identified. To highlight predominant orders within each protein group, sequences representing less than 5% of the total sequences from Ascomycota or Basidiomycota were grouped as “others,” separately for each phylum. Taxonomic assignments were based on UniProtKB annotations (column U in Table S2).

The proteins analyzed were widely distributed across the Fungal Kingdom, spanning two phyla, seven subphyla, 20 classes, 63 orders, and 175 families. Overall, the data set captured extensive phylogenetic and ecological diversity, encompassing fungi with contrasting lifestyles, including saprophytes involved in organic matter decomposition, plant and animal pathogens, endosymbionts, and mutualistic symbionts, such as mycorrhizal fungi. This diversity highlights the central role of acid phosphatases in phosphorus mobilization, nutrient acquisition, and adaptation to environmental constraints (Fig. 1 and 2 and Table S2). Despite this taxonomic breadth, the data set was strongly dominated by *Ascomycota*, which accounted for approximately 92% of the sequences used to construct the phylogenetic tree, while only 8% originated from *Basidiomycota*. Because UniProtKB primarily contains manually curated sequences (51), this imbalance likely reflects biases introduced by application-oriented studies toward economically and or biomedical relevant taxa. In fact, Ascomycetes include numerous saprotrophic fungi efficient at exploiting recalcitrant organic substrates, as well as endophytes, plant-associated fungi, and human or plant pathogenic strains that frequently inhabit nutrient-poor environments where phosphorus availability limits growth. In this context, extracellular acid phosphatases play a key role in scavenging organic phosphate esters, thereby conferring a competitive advantage. Within *Ascomycota*, *Eurotiales* was the most represented order, contributing 580 protein sequences. The most frequent genera within this group were *Aspergillus* (71%), *Penicillium* (17%), and *Talaromyces* (5.2%), which are well-known for their saprophytic lifestyles, metabolic versatility, and capacity to thrive in oligotrophic or stressed environments. These genera are also widely exploited in biotechnology and industry, which have probably contributed to their overrepresentation (52). In contrast, other fungal genera, such as *Penicilliopsis* and *Thermomyces*, were only sparsely represented. Other well-represented fungal orders included *Hypocreales* (508 proteins, e.g., *Trichoderma* spp., *Fusarium* spp., *Beauveria* spp., *Cordyceps* spp., *Metarhizium* spp.), Pleosporales (234 proteins, e.g., *Alternaria* spp., *Cochliobolus* spp., *Pyrenophora* spp., *Ascochyta* spp.), and *Saccharomycetales* (233 proteins, e.g., *Candida* spp., *Saccharomyces* spp., *Lachancea* spp., *Kluyveromyces* spp., *Pichia* spp., *Hanseniaspora* spp.). These genera encompass a wide spectrum of ecological strategies, ranging from saprotrophs and endophytes to aggressive phytopathogens, insect pathogens, and opportunistic human pathogens. The recurrence of acid phosphatases across these lineages further highlights their functional importance in nutrient acquisition, host interaction, and ecological success within fungi with different lifestyles and niches (12, 53, 54).

All eight groups of fungal phosphatases were found within *Ascomycota*, although unevenly distributed. In *Eurotiales*, *Hypocreales,* and *Pleosporales*, all the groups were identified except Group VI. In contrast, 96% of *Saccharomycetales* sequences were grouped in Group III, while other groups (I and VIII) were minimally represented, and Groups II, IV, V, and VI, and VII were absent (Fig. 2).

Within *Basidiomycota*, the most abundant orders were *Ustilaginales* (e.g., *Ustilago* spp., *Sporisorium* spp., *Pseudozyma* spp.) accounting for 48 protein sequences (20%). Members of this order are predominantly plant-associated biotrophs, including economically important smut pathogens of grasses and cereals, although some taxa exhibit yeast-like saprotrophic or epiphytic lifestyles (55). This was followed by Polyporales (e.g., *Trametes* spp., *Sparassis* spp., *Dichomitus* spp.) with 42 proteins (18%), an order largely composed of wood-decaying saprotrophs that play a central role in lignocellulose degradation and nutrient recycling (56). Agaricales (e.g., *Amanita* spp.*, Armillaria* spp., *Laccaria* spp., *Leucoagaricus* spp.) contributed 33 protein sequences (14%) and encompassed a broad range of ecological strategies, including ectomycorrhizal symbionts, saprotrophs, and plant pathogens. Tremellales (*Cryptococcus* spp., *Kwoniella* spp., *Naematelia* spp.) with 30 protein sequences (13%), primarily included yeast-forming saprotrophs, opportunistic animal-associated pathogens, and species with mycoparasitic tendencies. Other orders, such as *Wallemiales*, *Malasseziales*, and *Boletales,* were less represented, comprising 0.4%–6% of *Basidiomycota*-derived sequences. These groups included fungi adapted to extreme or specialized niches, such as xerophilic saprotrophs (*Wallemiales*), lipid-dependent animal-associated yeasts (*Malasseziales*), and predominantly ectomycorrhizal symbionts (*Boletales*) (57). Interestingly, within *Basidiomycota,* no sequences were assigned to Groups II and III, while Groups IV, V, and VI were poorly represented. The majority of sequences clustered in Groups VII (41%), VIII (32%), and I (21%) (Fig. 2). Although these patterns are shaped by taxonomic biases in publicly available databases, they provide a valuable framework for future studies investigating the functional diversification and ecological significance of acid phosphatases in *Basidiomycota*. Furthermore, these sets of results support the hypothesis that constructing fungal acid phosphatase profiles and applying them to metagenomic data sets may facilitate the discovery of previously uncharacterized enzymes that enhance our knowledge of their roles in P-cycling within ecosystems.

### Construction and validation of PROSITE generalized profiles for fungal acid phosphatases

To date, no protein profiles specific to fungal phosphatases have been described. As a first step toward distinguishing the different groups identified in the phylogenetic tree, we constructed PROSITE generalized profiles, which are not available in the PROSITE database (https://prosite.expasy.org/), following the approach previously used for bacteria phosphatases in Udaondo et al. (28).

The construction of protein profiles involves converting multiple sequence alignments into weighted matrices that assign numerical values to matches, mismatches, insertions, and deletions at each position. These values are summed and compared against a threshold (58) to determine significance. A Z-score threshold of ≥8.5 has been widely used in previous studies employing PROSITE generalized profiles to define significant matches (28, 58–60), although higher thresholds are sometimes used to enhance specificity (e.g., 60).

**Fig 3:**
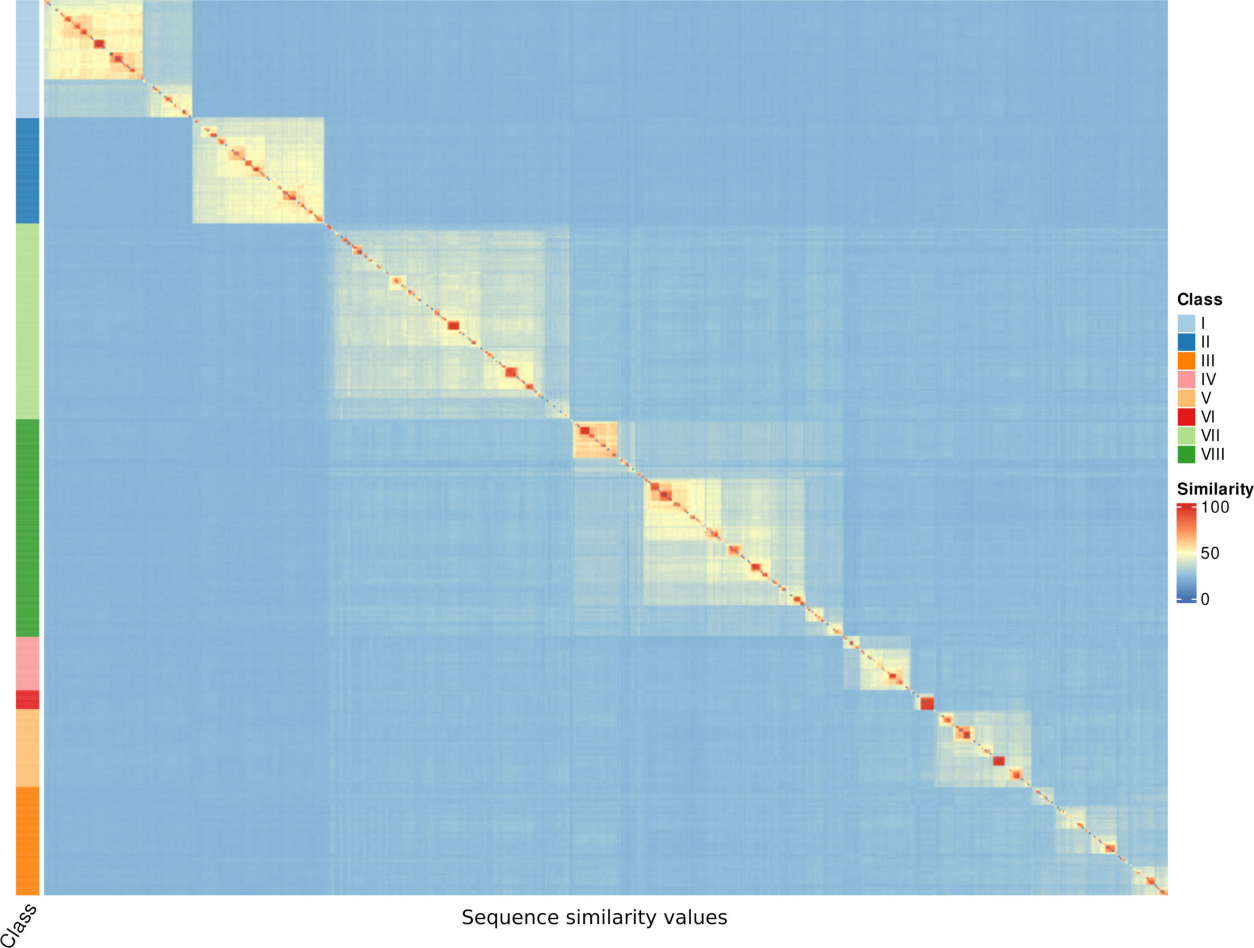
Heatmap of sequence similarity among fungal phosphatases. Each colored cell represents the degree of sequence similarity between protein pairs, illustrating clustering patterns that support protein profile definition: Prf-A-Fungal_phos (Group I), Prf-B-Fungal_phos (Group II), and Prf-C-Fungal_phos (Groups from III to VIII).

PROSITE generalized profiles were constructed using full-length sequences representative of each of the eight fungal phosphatase groups identified in the phylogenetic tree. Following the procedures described in Materials and Methods, one profile was generated for each group. These analyses led to the development of distinct and specific profiles for Group I (referred to as Prf-A-Fungal_phos) and Group II (referred to as Prf-B-Fungal_phos), each of which exclusively recognized sequences from its respective group. In contrast, profiles generated for Groups III to VIII lacked sufficient discriminatory power to distinguish these groups individually. However, none of these profiles cross-recognize protein sequences from Groups I or II. This observation aligns with the phylogenetic tree, which shows that proteins from Groups III to VIII are more closely related to each other than to those of Groups I and II. The greater sequence homology among Groups III to VIII probably accounts for the limited group-level resolution in their profiles (Fig. 3). Thus, we were prompted to construct a broader profile encompassing sequences from Groups III to VIII, which were developed and referred to as Prf-C-Fungal_phos. Heatmap analyses based on sequence similarity clustering further supported this combined profile, showing that proteins from Groups III to VIII cluster apart from those from Groups I and II (Fig. 3).

When applied to the complete set of 3,058 protein sequences from the phylogenetic tree, Prf-A-Fungal_phos identified all 403 Group I proteins, with Z-scores ranging from 21.70 to 80.04 (Table 2 and Table S3); Prf-B-Fungal_phos retrieved 360 Group II proteins, with Z-score values ranging from 51.83 to 73.56 (Table 2 and Table S4); and finally, Prf-C-Fungal_phos, which includes more heterogeneous sequences, retrieved 2,295 proteins from Groups III to VIII, with Z-scores ranging from 10.43 to 33.43, matching the total number of proteins in these groups (Table 2 and Table S5). Notably, no false positives (i.e., sequences incorrectly matched to a profile) or false negatives (i.e., sequences that should have matched but were not detected) were observed. This indicates high specificity of all three profiles within the curated data set, which together comprehensively covered all sequences used in the phylogenetic tree (Table 2).

**TABLE 2:** Number of sequences recognized by each of the constructed protein profiles for fungal phosphatases within each group of sequences displayed in the phylogenetic tree^*a*^

Profile	I	II	III	IV	V	VI	VII	VIII	Total	Z-score (min–max)
A	403	0	0	0	0	0	0	0	403	21.699–80.035
B	0	360	0	0	0	0	0	0	360	51.830–73.564
C	0	0	370	183	266	64	669	743	2,295	10.426–33.427

^*a*^ The range of Z-score values of sequences retrieved is also indicated. Raw information of the list of hits found by each profile including detailed Z-score value is provided in Table S3 (A, Prf-A-Fungal_phos), Table S4 (B, Prf-B-Fungal_phos), and Table S5 (C, Prf-C-Fungal_phos).

**Fig 4:**
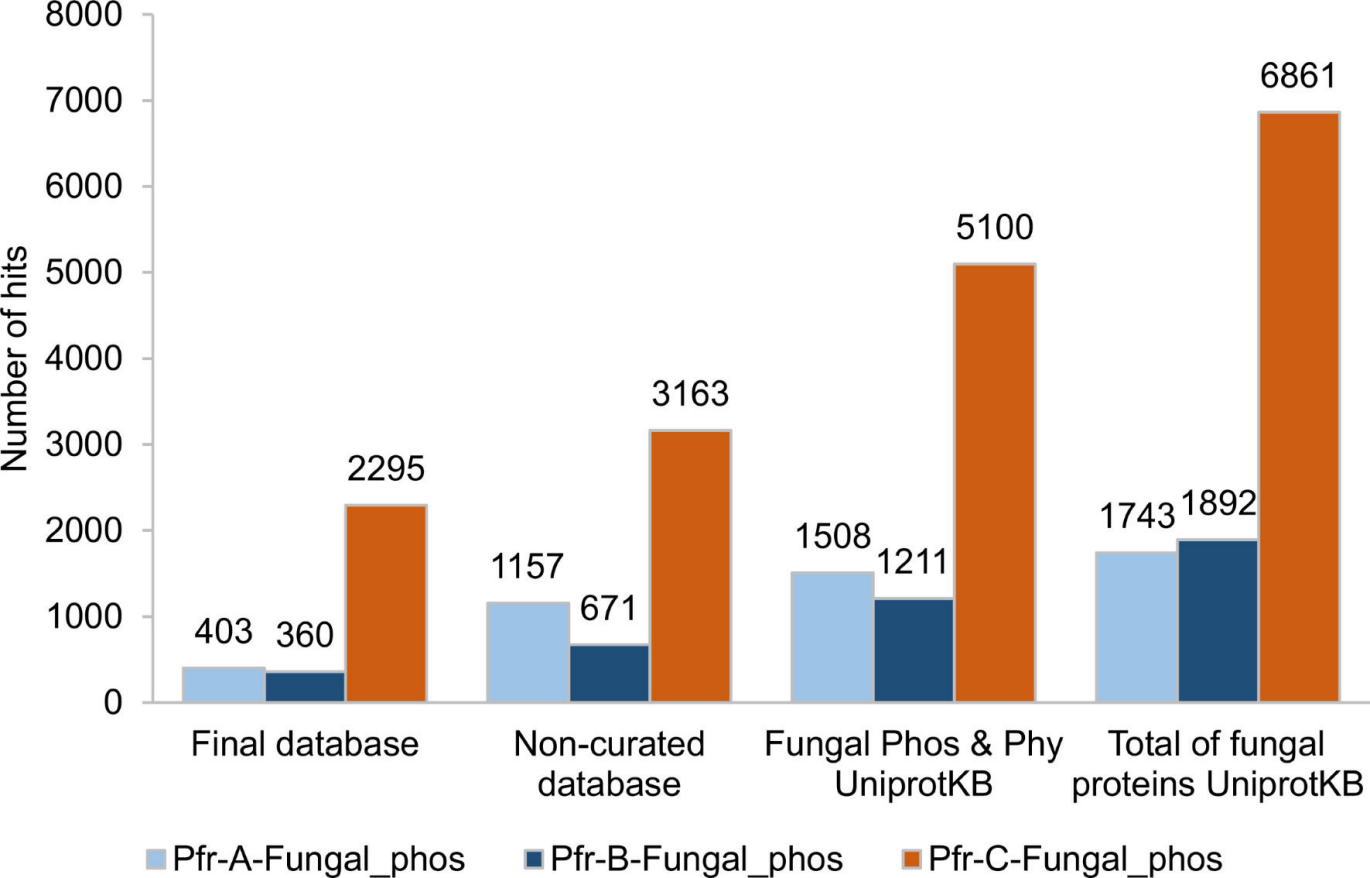
Number of hits detected by each constructed profile based on UniProtKB data sets. The number of fungal phosphatase sequences identified by the three profiles (Prf-A-Fungal_phos; Prf-B-Fungal_phos; Prf-C-Fungal_phos) for fungal phosphatases in UniProtKB data sets is shown. The data sets include (i) “Final database,” comprising 3,058 protein sequences used to construct the phylogenetic tree. (ii) “Non-curated database,” containing 8,859 non-reviewed fungal phosphatase sequences; (iii) “Fungal Phos & Phy UniProtKB,” including 20,050 fungal sequences annotated as “acid phosphatase” or “phytase” in UniProtKB database; and (iv) “Total of fungal proteins UniProtKB,” encompassing the complete collection of 19,274,552 fungal protein sequences. Detailed protein entries and Z-score for each profile in the corresponding data sets are provided in Table S3 (Prf-A-Fungal_phos), Table S4 (Prf-B-Fungal_phos) and Table S5 (Prf-C-Fungal_phos).

In order to further assess their sensitivity and validate the profiles, each was tested against larger UniProtKB data sets as detailed in Materials and Methods. We found that the number of sequences retrieved increased for each profile, yet specificity and discriminatory power were maintained, even for those with lower Z-scores, which supports the robustness of the three profiles (Fig. 4 and Tables S3 to S5). The higher number of hits in the UniProtKB data sets was attributed to their ability to detect incomplete or non-annotated sequences that nonetheless contained the hallmark domains of the corresponding groups (Tables S3 to S5).

**Fig 5:**
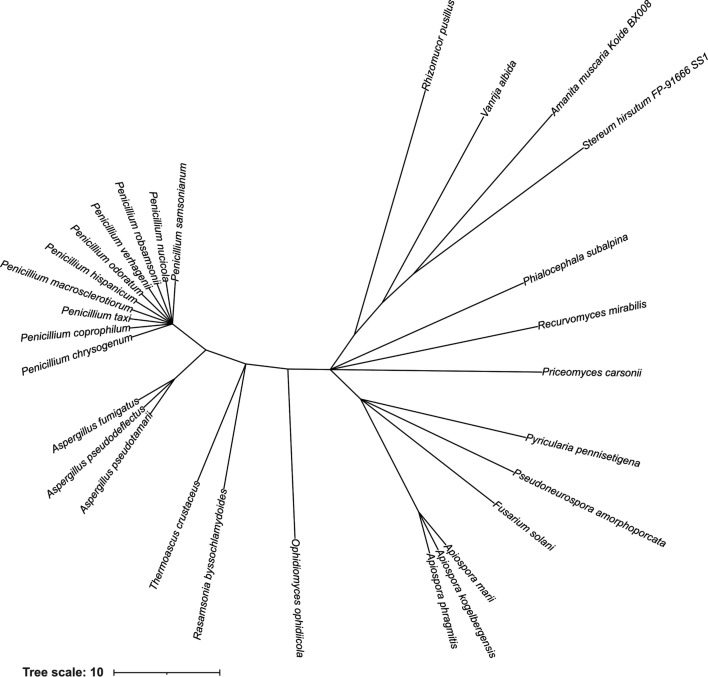
Taxonomic distribution of candidate phosphatases retrieved with Prf-A-Fungal_phos. Proteins annotated as “hypothetical protein,” “unknown protein,” or “uncharacterized protein” in UniRef90 and retrieved usingthe Prf-A-Fungal_phos profile are shown. Taxonomic identifiers from matched sequences were mapped and visualized using the NCBI Taxonomy Browser to illustrate the phylogenetic diversity of the candidate phosphatases identified by this profile.

Additionally, the three fungal phosphatase profiles were used to search a filtered UniRef90 database comprising 29,638,836 clusters of proteins annotated as hypothetical, unknown or uncharacterized proteins. The Prf-A-Fungal_phos profile identified 44 uncharacterized protein sequences across a broad taxonomic spectrum with Z-scores ranging from 8.50 to 77.73. These sequences were taxonomically diverse, spanning both *Ascomycota* and *Basidiomycota*. Within *Ascomycota*, many hits were affiliated with genera such as *Penicillium*, *Aspergillus*, *Apiospora*, and *Rhizomucor*, which are known for their roles in soil, plant association, or industrial applications. *Penicillium* was particularly well represented, accounting for more than one-third of the hits. Sequences were also retrieved from less commonly studied genera such as *Recurvomyces*, *Ophidiomyces*, and *Priceomyces*. In *Basidiomycota*, the profile retrieved sequences from *Amanita muscaria* and *Stereum hirsutum*, highlighting the ability of the Prf-A-Fungal_phos profile to detect distantly related phosphatases. The taxonomic distribution of the uncharacterized proteins retrieved with this profile is shown in Fig. 5 and Table S6.

**Fig 6:**
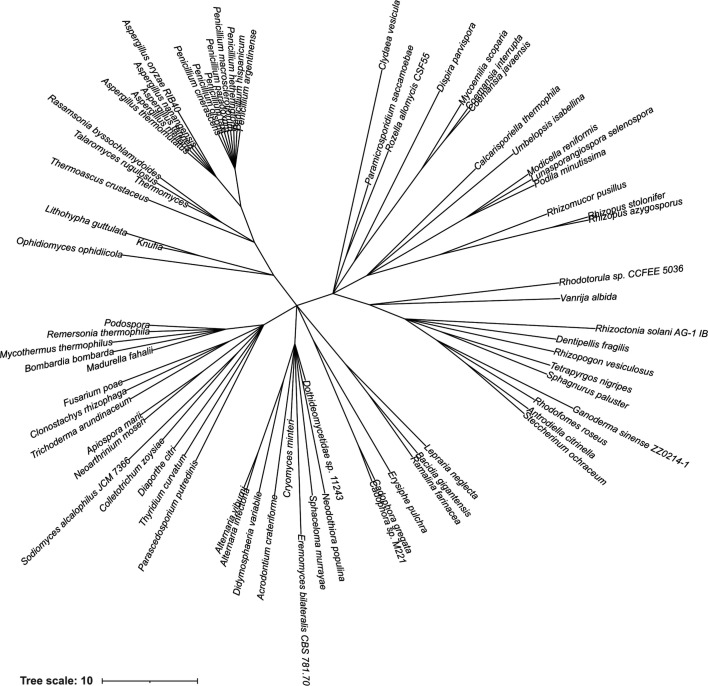
Taxonomic distribution of candidate phosphatases retrieved with Prf-B-Fungal_phos. Proteins annotated as “hypothetical protein,” “unknown protein,” or “uncharacterized protein” in UniRef90 and retrieved using the Prf-B-Fungal_phos profile are shown. Taxonomic identifiers from matched sequences were mapped and visualized using the NCBI Taxonomy Browser to illustrate the phylogenetic diversity of the candidate phosphatases identified by this profile.

The Prf-B-Fungal_phos profile retrieved 104 fungal protein sequences from the UniRef90 database, annotated as uncharacterized, hypothetical, or unknown proteins with Z-scores ranging from 8.51 to 70.41. We were able to identify candidate phosphatase sequences from both major fungal phyla, *Ascomycota* and *Basidiomycota*, as well as from early-diverging lineages, such as *Rozellomycota*, *Mucoromycota*, and *Zoopagomycota*. Notably, sequences were identified in species from diverse ecological niches, including plant pathogens (e.g., *Rhizoctonia solani*), symbionts (e.g., *Rozella allomycis*), endophytes, and thermophilic fungi (Fig. 6 and Table S7).

**Fig 7:**
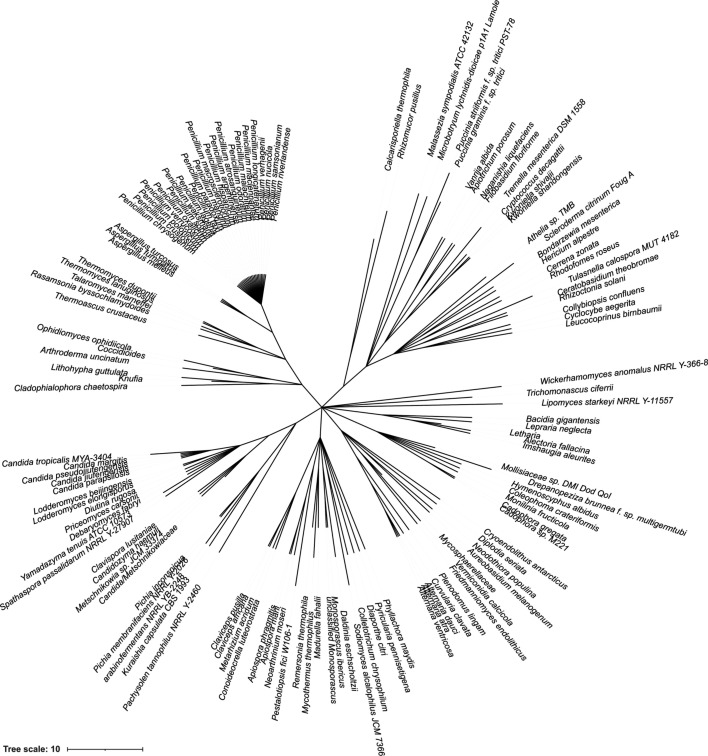
Taxonomic distribution of candidate phosphatases retrieved with Prf-C-Fungal_phos. Proteins annotated as “hypothetical protein”, “unknown protein,” or “uncharacterized protein” in UniRef90 and retrieved using the Prf-C-Fungal_phos profile are shown. Taxonomic identifiers from matched sequences were mapped and visualized using the NCBI Taxonomy Browser to illustrate the phylogenetic diversity of the candidate phosphatases identified by this profile.

The Prf-C-Fungal_phos profile retrieved a total of 293 fungal protein sequences from the UniRef90 database, all annotated as hypothetical, uncharacterized, or unknown proteins, with Z-scores ranging from 8.53 to 19.63. This profile captured the broadest taxonomic range among the three, reflecting its design to detect homologs across phosphatase Groups III to VIII. The majority of sequences (over 80%) belonged to the phylum *Ascomycota*, with *Penicillium* once again standing out as the most represented genus. Other frequently identified genera included *Aspergillus*, *Talaromyces*, *Fusarium*, and *Trichoderma*, all known for their ecological and industrial significance. Within *Basidiomycota*, the profile successfully detected proteins from species, such as *Schizophyllum commune*, *Fomitopsis pinicola*, and *Rhizoctonia solani*, highlighting its utility in recognizing acid phosphatase homologs in both saprophytic and pathogenic lineages (Fig. 7 and Table S8).

These results highlight the potential utility of the designed fungal acid phosphatase profiles for uncovering a previously uncharacterized and widespread members of acid phosphatase families in fungi. The wide taxonomic distribution of the lineages captured for the three profiles demonstrates their capacity to detect distantly related and potentially novel acid phosphatase candidates from across the fungal tree of life. The diversity of identified taxa, spanning from industrial workhorses to cryptic soil fungi and obligate symbionts, emphasizes the broad functional relevance of acid phosphatases.

### Experimental validation of fungal phosphatase profiles

**Fig 8:**
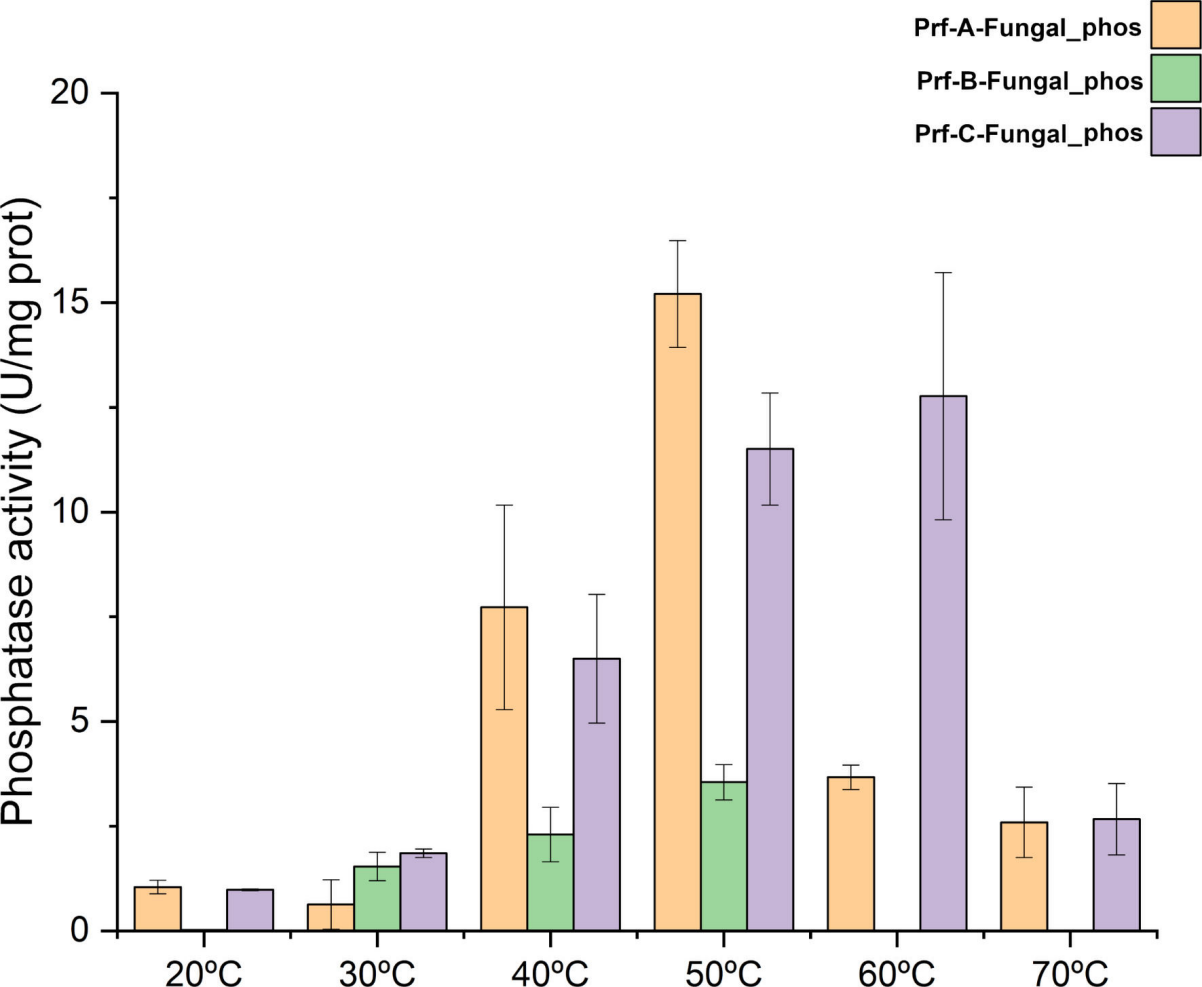
Phosphatase activity of synthetic fungal acid phosphatases. Phosphatase activity assays of proteins expressed from synthetic genes cloned in pDR196 (profiles Prf-A-Fungal_phos and Prf-C-Fungal_phos) or pYES2 (Prf-B-Fungal_phos). Synthetic genes were synthesized *in vitro* and transformed into *S. cerevisiae* BY4741. Enzymatic activity was measured using the standard assay, except that the temperature was varied as indicated. Data represent the mean ± standard deviation from three independent experiments.

As a proof of concept and to empirically validate the predicted profiles, one non-reviewed protein sequence with a high Z-score was selected from each profile: *Hyaloscypha variabilis* from Prf-A-Fungal_phos, *Verticillium alfalfa*e from Prf-B-Fungal_phos, and *Ascochyta rabiei* from Prf-C-Fungal_phos. The corresponding DNA sequences were synthesized and codon-optimized for expression in *Saccharomyces cerevisiae*, and subsequently cloned into the pDR196 expression vector (Profiles Prf-A-Fungal_phos and Prf-C-Fungal_phos) or into pYES2 (Prf-B-Fungal_phos). This alternative vector was used because preliminary expression trials indicate low or undetectable activity of *V. alfalfa*e acid phosphatase when cloned into pDR196. All three constructs were transformed and expressed in *S. cerevisiae* strain BY4741. Cells were grown to late exponential phase under constitutive expression in pDR196 or induced with galactose for 24h in pYES2. Following harvesting, cell-free extracts were prepared as described in Materials and Methods, and both acid phosphatase and phytase activities were assayed. The results (Table S9) confirmed that all three proteins exhibited phosphatase activity at 37°C and pH 5.5 with pNPP as a substrate, with specific activities ranging from 2.31 ± 0.65 to 7.90 ± 1.31 U/mg protein. No activity was detected at alkaline pH (i.e., 8.5), supporting their functional annotation of the previously uncharacterized sequences as acid phosphatases. No phytase activity was detected within this limited enzyme set. Notably, proteins detected and assayed from Prf-A-Fungal_phos, Prf-B-Fungal_phos, and Prf-C-Fungal_phos exhibited maximal activity at acidic pH at 50°C, although lower levels of activity were also measured at 60°C (Fig. 8). Acid phosphatases assayed from the three synthetic enzymes showed no significant activity at 70°C, suggesting a modest thermophilic character. Sternke et al. (61) demonstrated that consensus proteins tend to accumulate structural features that enhance stability and are frequently more thermostable than their natural counterparts. In line with this, García-Franco et al. (62) showed that a fungal-derived *trans*-cinnamic acid decarboxylase with a high Z-score accumulated protein-stabilizing features that were not directly linked to catalytic activity. Hence, the modest thermophilic character observed for the high Z-score acid phosphatases described here is probably attributable to structural stabilization rather than intrinsic catalytic adaptation. To complete the initial characterization of the synthetic proteins, we determined their apparent affinity for pNPP at 50°C. These measurements revealed affinities in the low millimolar range, consistent with values reported for moderately thermophilic *Aspergillus* and *Trichoderma* acid phosphatases (63, 64) (Table S9).

### Ecological implications and biotechnological application of the profiles

Phosphorus limitation constrains productivity in both managed and natural soils, largely because much of the soil P pool is present in organic or poorly soluble forms inaccessible to plants and microbes. Phosphatases alleviate this limitation by mineralizing organic phosphorus compounds, thereby sustaining primary productivity and improving nutrient-use efficiency. The environmental costs associated with the intensive use of nitrogen, phosphorus, and potassium (NPK) fertilizers (65), including soil degradation, eutrophication, greenhouse gas emission, and long-term loss of microbial diversity, have intensified the need for biologically driven, sustainable alternatives (65, 66). In this context, enzyme-based biological fertilizers represent a promising approach to enhance *in situ* nutrient mobilization while reducing reliance on chemical inputs (67–69).

Soil phosphorus bioavailability is shaped by physicochemical factors such as pH, mineral composition, and organic matter content, as well as by microbial activity and land management. Inorganic phosphorus is often immobilized through precipitation with calcium, iron, or aluminum. Microbes can solubilize these compounds by excreting weak and strong acids to release orthophosphate that can be assimilated by plants and microbes. Organic phosphorus, representing up to 50% of total soil phosphorus (70, 71), derives from plant and animal residues as well as microbial biomass (70, 72). Phytic acid often dominates the organic phosphorus pool and tends to accumulate in undisturbed or virgin soils, where it forms insoluble metal complexes that limit its availability to plants (71). Microbial biomass phosphorus, largely derived from nucleic acids (73), is often used as an informative indicator of bioavailable soil phosphorus (74).

The three validated PROSITE profiles developed here, resolve fungal acid phosphatases into distinct, phylogenetically coherent groups, enabling the identification of functional guilds rather than treating phosphatase activity as a uniform microbial trait. This stratification reflects evolutionary diversification in phosphorus acquisition strategies and may reflect adaptation to contrasting environmental conditions, such as soil pH, temperature, and organic matter composition. Biochemical differences among profile-associated enzymes suggest that different guilds contribute to phosphorus cycling under different ecological niches.

The broad taxonomic coverage of these profiles, including saprotrophs, plant-associated fungi, pathogens, endophytes, and symbionts across Ascomycota and Basidiomycota, highlights the conserved and widespread role of acid phosphatases in fungal ecology. In natural soils, these enzymes contribute to litter decomposition, organic matter turnover, and nutrient recycling in natural soils. In agricultural systems, they play a key role in rhizosphere phosphorus mobilization, influencing crop nutrition and fertilizer-use efficiency (25, 29, 31, 75).

The coexistence of multiple phosphatase guilds within soil fungal communities is consistent with functional complementarity and redundancy, potentially enhancing the resilience of phosphorus cycling under environmental disturbance, land-use change, or climatic variability (76, 77). At the same time, functional specialization implies that shifts in soil conditions or management practices may selectively favor particular guilds, with consequences for phosphorus availability and ecosystem functioning.

Beyond these ecological insights, the profiles developed in this study provide a powerful tool for the functional annotation of genomic, metagenomic, and meta-transcriptomic data sets (28, 78), particularly in poorly characterized soil fungi. Their high specificity and initial experimental validation strengthen the link between sequence-based predictions and enzymatic function, facilitating trait-based ecological analyzes and improving models of nutrient cycling. From an applied perspective, profile-guided phosphatase discovery opens new avenues for sustainable phosphorus management, including the identification of candidate enzymes for the development of biofertilizers, enzyme-based soil amendments, and management strategies aimed at enhancing biological phosphorus mobilization while reducing dependence on mineral fertilizers.

Together with previously developed bacterial phosphatase profiles (28), the tools presented here expand the capacity to uncover novel enzymes and improve our understanding of phosphorus dynamics. Across terrestrial and aquatic ecosystems. These profiles not only facilitate the identification of previously uncharacterized phosphatases but also open new avenues for biotechnological applications in phosphorus recovery, particularly in nutrient-poor soils and contaminated environments. As the demand for sustainable agricultural practices and efficient nutrient management continues to grow, the development and application of novel, and biotechnologically optimized acid phosphatases may contribute to improved soil fertility and more sustainable phosphorus management. By enhancing biological phosphorus mobilization, such approaches could help reduce dependence on mineral fertilizers and support broader efforts toward resource efficiency and environmental sustainability.

## Data Availability

Protein profiles designed in this study were deposited in the Zenodo database and can be accessed at https://doi.org/10.5281/zenodo.16876021. In addition, data sets analyzed during the present study are provided as supplemental material.

## SUPPLEMENTAL MATERIAL

Supplemental methods aem.02106-25-s0001.docx Strains and plasmids, phytase activity, and Table S9.

Table S1 aem.02106-25-s0002.docx Primers used in experimental procedures.

Table S2 aem.02106-25-s0003.xlsx Metadata corresponding to the final set of fungal protein sequences annotated as "acid phosphatase" or "phytase" in UniProtKB database used to construct the phylogenetic tree.

Table S3 aem.02106-25-s0004.xlsx List of hits found by Pfr-A-Fungal_phos profile on the different datasets downloaded from UniprotKB and sorted by their Z-score value.

Table S4 aem.02106-25-s0005.xlsx List of hits found by Pfr-B-Fungal_phos profile on the different datasets downloaded from UniprotKB and sorted by their Z-score value.

Table S5 aem.02106-25-s0006.xlsx List of hits found by Pfr-C-Fungal_phos profile on the different datasets downloaded from UniprotKB and sorted by their Z-score value.

Table S6 aem.02106-25-s0007.xlsx Metadata collected from uncharacterized fungal proteins identified by Pfr-A-Fungal_phos.

Table S7 aem.02106-25-s0008.xlsx Metadata collected from uncharacterized fungal proteins identified by Pfr-B-Fungal_phos.

Table S8 aem.02106-25-s0009.xlsx Metadata collected from uncharacterized fungal proteins identified by Pfr-C-Fungal_phos.

ASM does not own the copyrights to Supplemental Material that may be linked to, or accessed through, an article. The authors have granted ASM a non-exclusive, world-wide license to publish the Supplemental Material files. Please contact the corresponding author directly for reuse.
