# Supplementary material for: Development of advanced bioinformatic profiles to improve the detection and functional understanding of fungal acid phosphatases
Source: Appl Environ Microbiol. 2026 Jun 12;92(7):e02106-25. doi: 10.1128/aem.02106-25 (PMC13390437; doi:10.1128/aem.02106-25)
Supplement: Supplemental methods — Strains and plasmids, phytase activity, and Table S9. [file aem.02106-25-s0001.docx]

**SUPPLEMENTARY MATERIAL**

**Development of Advanced Bioinformatic Profiles to Improve the Detection and Functional Understanding of Fungal Acid Phosphatases**

Támara Gomez-Gallego^a^, Zulema Udaondo^b#^, Rocio Palacios-Ferrer^a,d^, Luis Díaz-Martínez^c^ & Juan L. Ramos^a^

*^a^* Department of Environmental Protection, Estación Experimental del Zaidín, Consejo Superior de Investigaciones Científicas (CSIC), Granada, Spain.

^b^Department of Microbial Biotechnology, National Center for Biotechnology, Consejo Superior de Investigaciones Científicas (CSIC), Campus de la Universidad Autónoma de Madrid, Canto Blanco, Madrid, Spain

^c^ Departamento de Microbiología, Instituto de Hortofruticultura Subtropical y Mediterránea ‘La Mayora’, Universidad de Málaga-Consejo Superior de Investigaciones Científicas (IHSM-UMA-CSIC), Universidad de Málaga, Málaga, Spain

^d^ Programa de Doctorado en Bioquímica y Biología Molecular, Universidad de Granada, Granada, Spain

**Materials and Methods**

**Strains and plasmids**

*Saccharomyces cerevisiae* strain BY4741 (MATa his3Δ1 leu2Δ0 met15Δ0 ura3Δ0) was used for heterologous gene expression. The strain was maintained on YPD agar plates (1% yeast extract, 2% peptone, 2% glucose, 2% agar).

The pRD196 plasmid carries the URA3 selectable marker for auxotrophic selection in *Saccharomyces cerevisiae*, and the 2μ origin for high-copy replication. It carries the PMα1 promoter, a constitutive promoter derived from the *PMA1* (plasma membrane H⁺-ATPase) gene. The plasmid also contains an ampicillin gene for selection in *Escherichia coli*, and a multiple cloning site for gene insertion. on synthetic defined (SD) medium lacking uracil at 30 °C with shaking at 200 rpm.

Transformation of yeasts. Briefly, a pre-culture of *S. cerevisiae* BY4741 was prepared by inoculating 20 mL of YPD medium (1% yeast extract, 2% peptone, 2% glucose) and incubating overnight at 30 °C with shaking at 200 rpm. The culture was then diluted into 50 mL of fresh YPD medium to an initial OD_600_ of 0.2–0.3 and grown for 2–4 h until reaching an OD_600_ of approximately 1.

Cells were harvested by centrifugation of 10 mL aliquots at 3,000 × g for 5 min, washed once with 8 mL of sterile distilled water, and centrifuged again under the same conditions. The resulting pellet was resuspended in 1 mL of 100 mM lithium acetate solution (LiAc) and transferred to microcentrifuge tubes. After centrifugation at 4,000 rpm for 5 min, cells were resuspended in 100 μL of 100 mM LiAc and kept on ice. Aliquots of 50 μL were prepared, centrifuged briefly at maximum speed (13,000–14,000 rpm), and the cell pellets were used immediately for transformation. For each transformation reaction, the cell pellet was mixed with 300 μL of 40% PEG 3350 solution, 6 μL of 1 M LiAc, 25 μL of denatured salmon sperm DNA (2 mg/mL, boiled and chilled), 1 μg of plasmid DNA, and sterile water to a final volume of 360 μL. The mixture was gently pipetted using a wide-bore tip and incubated at 30 °C for 30 min with shaking. Heat shock was performed at 42 °C for 20 min, followed by centrifugation at 7,000 rpm for 1 min. The supernatant was discarded, and cells were resuspended in 1 mL of sterile water and plated onto selective synthetic dropout (SD) agar medium.

**Phytase activity**

Phytase activity was determined as suggested by Qvirist et al. (2015). Briefly, A 150 microliters aliquot of 5 mM phytic acid solution was pre-incubated in HAM buffer for 5 minutes at 37°C. Then, 16.6 µL of resuspended pellet or supernatant was added, and the mixture was incubated for 30 minutes at 37°C. To stop the reaction, 1.33 ml of a 2:1:1 mixture of acetone:H_2_SO_4_:(NH_4_)_6_Mo_7_O_24_ was added. The resulting color was measured spectrophotometrically at 405 nm using a Tecan Sunrise plate reader (Tecan Austria GmbH).

| **Enzyme** | **Phosphatase activity (U/mg prot) , pH 5.5** | **K_m_^´^**  **^(mM)^** |
| --- | --- | --- |
| Prf-A-Fungal_phos | 4.88 ± 1.01 | 0.054 ± 0.002 |
| Prf-B-Fungal_phos | 3.59 ±0.56 | 0.150 ± 0.013 |
| Prf-C-Fungal_phos | 7.55 ±0.50 | 0.057 ± 0.013 |

Supplementary Table 9. Phosphatase activity of proteins expressed from synthetic genes cloned in pDR196. Genes were synthesized in vitro, transformed into *S. cerevisiae* BY4741 and assayed enzymatically as described in the experimental procedures. Values are given in the experimental procedures. The values represent the mean ± average standard deviation from three independent experiments.

**Supplementary Tables legends:**

**Table S1**: Primers used in experimental procedures.

**Table S2**: Metadata corresponding to the final set of fungal protein sequences annotated as “acid phosphatase” or “phytase” in UniProtKB database used to construct the phylogenetic tree. Searches in UniProtKB database yielded 8,859 putative phosphatase sequences that were analyzed and filtered as described in the material and methods section, resulting in a final set of 3,058 protein sequences.

**Table S3**: List of hits found by Pfr-A-Fungal_phos profile on the different datasets downloaded from UniprotKB and sorted by their Z-score value.

**Table S4**: List of hits found by Pfr-B-Fungal_phos profile on the different datasets downloaded from UniprotKB and sorted by their Z-score value.

**Table S5**: List of hits found by Pfr-C-Fungal_phos profile on the different datasets downloaded from UniprotKB and sorted by their Z-score value.

**Table S6:** Metadata collected from uncharacterized fungal proteins identified by the Pfr-A-Fungal_phos

**Table S7:** Metadata collected from uncharacterized fungal proteins identified by the Pfr-B-Fungal_phos

**Table S8:** Metadata collected from Uncharacterized fungal proteins identified by the Pfr-C-Fungal_phos

**Table S9:** Phosphatase activity of proteins expressed from synthetic genes cloned in pDR196. Genes were synthesized in vitro, transformed into *S. cerevisiae* BY4741 and assayed enzymatically as described in the experimental procedures. Values are given in the experimental procedures. The values represent the mean ± average standard deviation from three independent experiments.

**References**

Qvirist, L., Carlsson, N. G., & Andlid, T. (2015). Assessing phytase activity–methods, definitions and pitfalls. *Journal of Biological Methods (JBM)*, *2*(1).
