# Supplementary material for: Development of advanced bioinformatic profiles to improve the detection and functional understanding of fungal acid phosphatases
Source: Appl Environ Microbiol. 2026 Jun 12;92(7):e02106-25. doi: 10.1128/aem.02106-25 (PMC13390437; doi:10.1128/aem.02106-25)
Supplement: Table S1 — Primers used in experimental procedures. [file aem.02106-25-s0002.docx]

| **Strain** | **Description** | **Source** |
| --- | --- | --- |
| *Saccharomyces cerevisiae* BY4741 | (MATa his3Δ1 leu2Δ0 met15Δ0 ura3Δ0) | Winston et al., 1995; Brachmann et al., 1998 |
| *E. coli* DH5α | Host strain for gene cloning |  |
| **Plasmid** | **Description** | **Source** |
| pDR196 – high-copy yeast expression vector | PMA promoter, URA3 selection marker, ampicillin resistance | Available at our lab collection |
| **Primer** | **Sequence (5´-3´)** | **Use** |
| P1_FW  P1_RV  P3-8_FW  P3-8_RV  pDR196_FW  pDR196_RV | ATGGCCGCTTTGTCTACTTTCG  TTGAACAACAAATTGTTCGG  ATGAGAGACACCGTCATCTCTCG  CTTGTAACATTGGTTCCACAAACC  CACACATTCAAAAGAAAGAAAA  GCAAGGTAGACAAGCCGACAAC | Profile 1 transformants checking  Profile 1 transformants checking  Profile 3-8 transformants checking  Profile 3-8 transformants checking  Transformants cheking  Transformants checking |

Supplementary table 1. Strains, plasmids an primers used in this study
